# Serum Ferritin Is Associated with Metabolic Syndrome and Red Meat Consumption

**DOI:** 10.1155/2015/769739

**Published:** 2015-09-14

**Authors:** Avila Felipe, Echeverría Guadalupe, Pérez Druso, Martinez Carlos, Strobel Pablo, Castillo Oscar, Villaroel Luis, Mezzano Diego, Rozowski Jaime, Urquiaga Inés, Leighton Federico

**Affiliations:** ^1^Programa de Investigación de Excelencia Interdisciplinaria en Envejecimiento Saludable (PIEI-ES), Universidad de Talca, 3460000 Talca, Chile; ^2^Centro de Nutrición Molecular y Enfermedades Crónicas, Pontificia Universidad Católica de Chile, Casilla 114-D, 8331150 Santiago, Chile; ^3^Departamento de Nutrición, Diabetes y Metabolismo, Facultad de Medicina, Pontificia Universidad Católica de Chile, 8330024 Santiago, Chile; ^4^Departamento de Salud Pública, Facultad de Medicina, Pontificia Universidad Católica de Chile, 8330024 Santiago, Chile; ^5^Laboratorio de Hemostasia, Facultad de Medicina, Pontificia Universidad Católica de Chile, 8330024 Santiago, Chile

## Abstract

*Background and Aims*. Hyperferritinemia has been related with a wide spectrum of pathologies, including diabetes, cardiovascular disease, neurodegenerative disorders, and metabolic syndrome. The aim of this study was to investigate the association between hyperferritinemia and iron consumption. *Methods and Results*. Serum ferritin concentration was evaluated in 66 presumed healthy men, along with other clinical and biochemical markers of chronic diseases. A three-day food questionnaire was applied for nutrition information. Hyperferritinemia was a condition found in 13.4% of the volunteers analyzed. Significant correlations were found between serum ferritin concentration and metabolic syndrome parameters (HDL cholesterol, triglycerides, and fasting glucose) as well as an increase of the serum ferritin mean value with the number of risk factors of metabolic syndrome. Also, oxidative stress markers (carbonyl groups, AOPP, and glycated hemoglobin), hepatic damage markers (GGT, SGOT), and parameters related to insulin resistance (HOMA, blood insulin, and blood glucose) correlate significantly with serum ferritin. Volunteers had an excessive iron intake, principally by bread consumption. Analyses of food intake showed that red meat consumption correlates significantly with serum ferritin. *Conclusion*. Red meat consumption, metabolic syndrome, and chronic disease markers are associated with hyperferritinemia in a population of Chilean men.

## 1. Introduction

Excessive accumulation of iron in the body has been proposed as a risk factor for a wide spectrum of diseases including cardiovascular diseases, liver dysfunction, peripheral arterial disease, and neurodegenerative diseases, among others [1–3].

Iron overload is characterized physiologically by an increase in the ferritin levels [2]. Hence, serum ferritin concentration is used as a first indicator in the diagnosis of iron overload-related diseases [3, 4]. It has become increasingly evident that high levels of serum ferritin can be produced as a consequence of metabolic disorders, which have not been associated with iron overload [2, 5].

Elevated serum ferritin levels, in the absence of high transferrin saturation, have appeared during the last years as a common characteristic in some patients with metabolic syndrome [4, 6]. It has been reported that hyperferritinemia observed in subjects with metabolic syndrome is associated with insulin resistance and fatty liver but not with iron overload, determined in liver biopsies by quantitative phlebotomy [4]. However, the mechanistic link between hyperferritinemia and metabolic syndrome as well as the effects of this condition is mostly unknown [7].

Dietary intake has been found to be closely related with the prevalence and prevention of metabolic syndrome [8]. In this sense, it has been demonstrated that consumption of a Western dietary pattern promotes the incidence of metabolic syndrome [9]. In turn, the Mediterranean diet pattern has shown to reduce metabolic syndrome and its related medical complications [10, 11]. In particular, the consumption of specific foodstuffs and their macro- and micronutrients appear to play a central role in the prevalence of metabolic syndrome [12–14]. The consumption of red meat has been related with metabolic syndrome and overexpression of C-reactive proteins in women [12]. Therefore, it is interesting to determine whether there is a role of specific nutrient intake in the metabolic syndrome-related hyperferritinemia. The aim of this study was to investigate the association between iron consumption and hyperferritinemia.

In this work, we assessed the iron overload in terms of serum ferritin concentration and total iron binding capacity (TIBC), in a sample of 66 Chilean male volunteers. The results are discussed in terms of the relationship between serum ferritin and the condition of metabolic syndrome and chronic disease markers. We discuss the contribution of main food group and dietary micronutrient intake to the levels of serum ferritin.

## 2. Experimental Methods

### 2.1. Subjects

(1) Male volunteers were recruited from a heavy machinery maintenance and repair company for the mining industry, “Maestranza Diesel,” in Santiago, Chile. A number of 129 of 171 total subjects were free of exclusion criteria. Simple random sampling was chosen to select 66 subjects for this study. The Ethics Committee of the Faculty of Medicine at the Universidad Católica de Chile approved this study.

*Exclusion Criteria*. Diabetes mellitus, blood hypertension, and dyslipidemia under treatment were excluded. Persons that actively adhered to specific diets or had participated in a weight loss program in the previous 6 months as well as persons undergoing pharmacological treatment with drugs that modify lipid profiles, blood pressure, carbohydrate metabolism, plasma antioxidant capacity, and inflammation were also excluded. The diagnosis of the metabolic syndrome followed the adult treatment panel III criteria: (i) abdominal adiposity (defined as waist circumference >102 cm (men) or >88 cm (women)); (ii) low levels of serum high-density lipoprotein cholesterol (<40 mg/dL (men) or <50 mg/dL (women)); (iii) hypertriglyceridemia, 150 mg/dL or more; (iv) elevated blood pressure, 130/85 mmHg or more; and (v) impaired glucose homeostasis (fasting plasma glucose concentration of 100 mg/dL or more).

(2) Paired control of volunteers was defined by sex and age. Male blood donors (*n* = 67) were recruited from the blood bank of the hospital of the Universidad Católica de Chile (BB). Requirements for blood donation were to be healthy (feeling well that day, not having colds, coughs, or flu in the last one week, and not having fever in the last 3 weeks), be over 18 years old, and weighting at least 45 kg (100 lbs.). Subjects must not have donated blood in the previous two months.

### 2.2. Study Design

Evaluation of each volunteer was carried out covering four aspects: clinical, anthropometric, biochemical, and nutritional. For nutrient intake analyses, a 3-day weighed-food record was applied and diet compositions were calculated employing the food processor II computer program (ESHA Research, Salem, OR, USA).

### 2.3. Analytical Procedures

Venous blood samples were collected after a 12-hour fasting period into heparin, citrate, and anticoagulant-free BD Vacutainer tubes. Plasma, serum, erythrocytes, and leukocyte samples were stored, following common laboratory procedures until they were analyzed. Plasma was obtained from freshly drawn blood by centrifugation at 1000 ×g for 5 min.

Glucose, albumin, insulin, calcium, total cholesterol, HDL cholesterol, LDL cholesterol, triglycerides, SGOT, and GGT were measured in serum using a spectrophotometer autoanalyzer (Hitachi 917, Roche Diagnostics, Branchburg, NJ) with reagent kits purchased from the manufacturer. Glycated hemoglobin (HBA1C) was determined by means of the HPLC method using the system VARIANT II (BioRad Laboratories).

Serum ferritin concentrations were measured using the kits ADVIA Centaur (Siemens Healthcare and Diagnostics, Tarrytown, NY, USA) according to the instructions provided by the manufacturer. Total iron binding capacity (TIBC) was calculated as the sum of the serum iron plus the Unsaturated Iron Binding Capacity (UIBC), which were determined using the kits Fe iron COBAS (Roche diagnostics GMBH) and Unsaturated Iron Binding Capacity COBAS (Roche diagnostics GMBH), according to the instructions provided by the manufacturer. Advanced oxidation end products (AOPP) were determined according to Witko-Sarsat et al. [15], and carbonyl groups were determined spectrophotometrically quantifying the generation of the 2,4-dinitrophenylhydrazine [16].

### 2.4. Statistical Analysis

The chi-square test was used to compare normal and abnormal values of serum ferritin concentration and TBIC. Additionally, normality distribution test of Kolmogorov-Smirnov was applied for serum ferritin concentration and TBIC. Two-tailed, unpaired Student's* t*-test was used to compare the means of different variables between the MD and BB groups. Proportions were compared by means of the two-tailed, Fisher's exact test. Correlations between variables were analyzed by Pearson correlation. One-way ANOVA was used to compare mean serum ferritin concentrations in different groups of individuals, without, with 1 or 2, and with 3 or more metabolic syndrome risk factors.

## 3. Results

A sample of 66 Chilean male volunteers chosen at random from 129 eligible workers of a mining machinery industry “Maestranza Diesel” (MD) were evaluated in terms of anthropometric and biochemical parameters. The mean values of all biochemical analyses were in agreement with the normal range values (Table 1) except BMI, triglycerides, and serum ferritin, which present mean values above normal.

**Figure 1:**
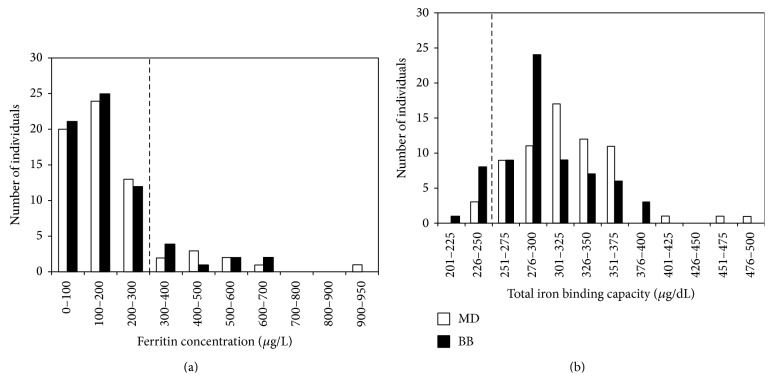
Serum ferritin (a) and TIBC (b) distribution in Maestranza Diesel workers (MD) and blood bank donors (BB); the number of individuals analyzed corresponds to *N* = 66 and *N* = 67, respectively. Dashed line indicates the cutoff between iron overload and normal iron levels, in terms of serum ferritin and total iron binding capacity, respectively.

With the purpose of assessing the iron status in this population, serum ferritin and TIBC were determined. Serum ferritin concentration ranged in subjects from 12.1 to 910.4 *μ*g/L, with a 13.6% of the total population (*N* = 66) with high levels of ferritin (>300 *μ*g/L) (Figure 1(a)). Only 4.5% of the volunteers presented iron overload, in terms of TIBC (<240), indicating that serum ferritin was the main altered parameter (Figure 1(b)).

In order to determine whether the results of this group of individuals were not related with their work in machinery maintenance and repair for the mining industry, we analyzed the prevalence of serum ferritin in 67 paired controls by sex and age, recruited from the blood bank of the hospital of the Universidad Católica de Chile. It was found that 13.6% of the volunteers from the blood bank had hyperferritinemia and 11.2% of the volunteers showed altered values of TIBC (Figures 1(a) and 1(b)).

Further analyses used to determine the origin of the observed hyperferritinemia were performed only with the MD population.

**Figure 2:**
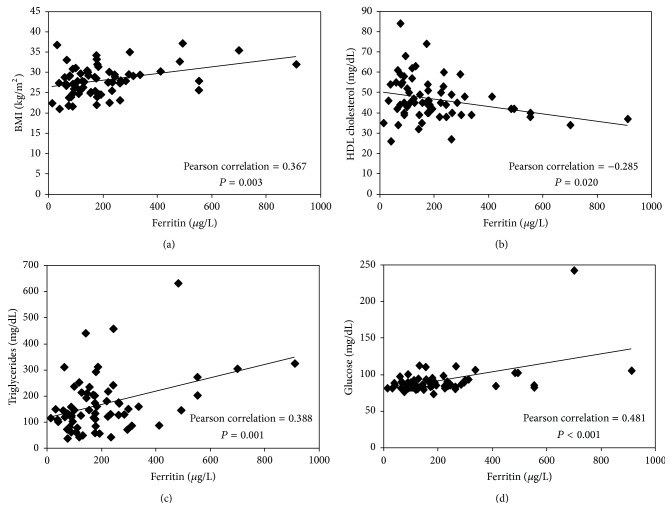
Significant correlation between serum ferritin levels and risk factors associated with the diagnostic of metabolic syndrome: BMI (a), HDL cholesterol (b), triglycerides (c), and fasting glucose (d).

Correlations were established between serum ferritin and metabolic syndrome related parameters to determine whether there is an association between these two conditions in MD workers (*N* = 66). Serum ferritin correlated significantly with BMI (*r* = 0.367; *P* = 0.003), HDL cholesterol (*r* = −0.285; *P* = 0.020), triglycerides (*r* = 0.388; *P* = 0.001), and fasting glucose (*r* = 0.481; *P* < 0.001) (Figure 2). Waist circumference and blood pressure did not correlate with serum ferritin.

**Figure 3:**
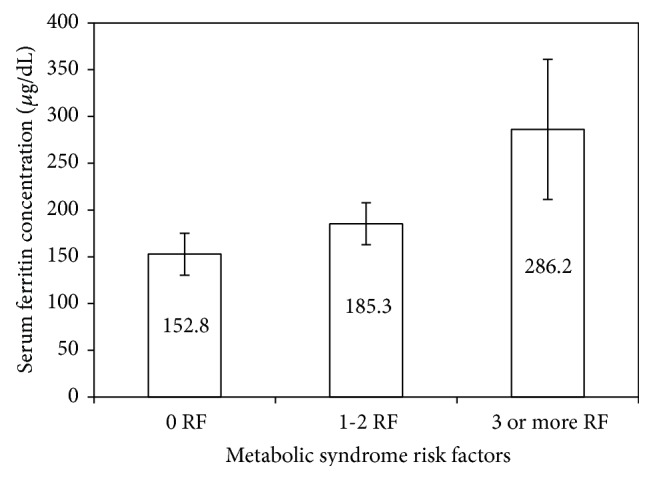
Variation of the mean value of serum ferritin concentration with the number of risk factors of metabolic syndrome according to ATPIII panel. ATP definition: waist circumference >102 cm (men); HDL cholesterol <40 mg/dL (men); triglycerides ≥150 mg/dL; blood pressure ≥130/85 mmHg; and fasting plasma glucose ≥100 mg/dL. The mean value of serum ferritin increases significantly with the number of risk factors related to metabolic syndrome (*P* = 0.043, one-way ANOVA). Results are expressed as means ± standard error (*n* = 20, *n* = 31, and *n* = 15 for individuals without, with 1 or 2, and with 3 or more risk factors of metabolic syndrome, resp.).

In order to analyze the relationship between metabolic syndrome and serum ferritin in MD subjects, the variation of the mean value of ferritin was determined for the total population, matching with increasing factors associated with metabolic syndrome diagnostic (according to ATP III). The mean value of serum ferritin increased significantly with the number of risk factors related to metabolic syndrome (Figure 3).

With the aim of determining whether serum ferritin could be associated with metabolic abnormalities leading to chronic diseases, correlations were established between serum ferritin and oxidative stress, hepatic damage, and insulin resistance related markers. A positive and statistically significant correlation between serum ferritin and oxidative stress markers (carbonyl groups, glycated HB, and AOPP), hepatic damage markers (GGT and SGOT), and insulin resistance related parameters (HOMA, blood insulin, and fasting glucose) was observed (Table 2).

In contrast, no significant correlations were observed between serum ferritin and inflammatory response markers such as C-reactive protein, fibrinogen, and leukocytes (Table 2).

The dietary intake of energy sources for volunteers was 2153 kcal on average (16.4% proteins, 28.4% fat, and 55.2% carbohydrates) and is consistent with WHO recommendations. However, selenium intake was 190% higher than the recommended values, whereas calcium was the mineral with the lowest intake with a value 37.3% lower than the recommended amount. The major vitamin over intake was given by vitamin B12, 110% higher than the recommended amount.

The contribution of the main food group intake responsible for the high micronutrient intake is shown in Table 3. The contribution of white bread to almost all micronutrient overloads is noteworthy, especially in iron (56.6%), sodium (52.1%), thiamine (73.3%), and riboflavin (60.7%).

The contribution of red meat to the high micronutrients intake also appears to be important, mainly in iron (13.9%), sodium (13.9%), selenium (25.5%), vitamin B12 (50.6%), and niacin (32.6%).

**Figure 4:**
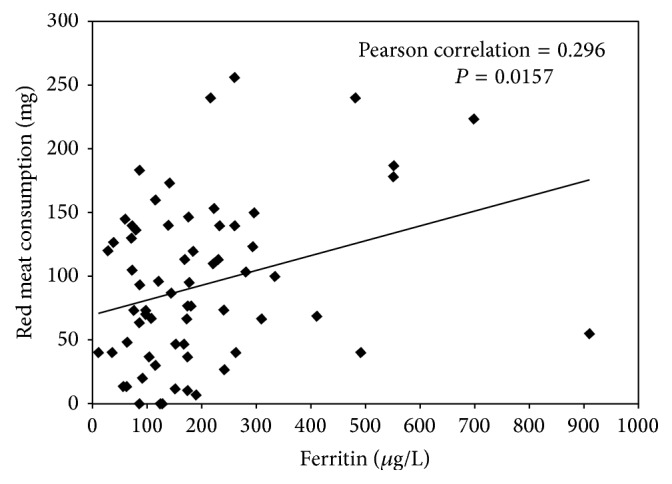
Correlation between serum ferritin concentration and red meat consumption. Red meat comprised all kinds of red meat including processed red meat. The amount of red meat corresponds to the average intake of three consecutive days.

In order to test if the observed hyperferritinemia is related with diet, correlations were performed between serum ferritin concentration and food sources showed in Table 3. It was found that only red meat and all kinds of red meat including processed red meat correlate positively and significantly with serum ferritin (Figure 4) (*r* = 0.296, *P* = 0.016).

## 4. Discussion

Although iron plays a fundamental role in many physiological processes, iron excess can lead to tissue damage mediated by free radicals through Fenton reaction [17]. The harmful effect of iron is decreased through binding to proteins, such as ferritin, which is involved in the iron homeostasis and acts as an iron storage protein [3].

Serum ferritin concentration is directly related to iron levels in the organism, and for this reason the evaluation of this protein in blood has been used as a primary diagnosis indicator in iron overload-related diseases [3, 4]. However, ferritin levels can also be altered in inflammation, in chronic renal insufficiency, and by metabolic disorders [18, 19]. For this reason, transferrin saturation is used as a complement in the iron overload diagnosis [18].

During the last years, increasing evidence has associated metabolic syndrome with hyperferritinemia [20]. Nevertheless, the relationship between serum ferritin and iron overload is still controversial, while some studies indicate a causative relationship of the effects of iron overload in several metabolic syndrome complications [7, 21]. There are some studies that indicate normal levels of iron [4] and a conserved iron regulatory feedback in metabolic syndrome [22]. The origins of hyperferritinemia associated with metabolic syndrome are not understood as well as the effects involved with this condition [22].

In this work, we show evidence from a group of 66 male volunteers indicating that serum ferritin levels are associated with metabolic syndrome and chronic disease parameters. In addition, we provide evidence that suggests that red meat consumption could be associated with this upregulation.

The evaluation of biochemical profiles in a sample of 66 volunteers indicates that serum ferritin was the main altered parameter (Table 1), with a 13.4% of the total population with high levels of ferritin (>300 *μ*g/L).

In order to determine whether the group of subjects chosen in this study can be considered as a representative sample of the Chilean adult male population and the results were not related with their specific work in machinery maintenance and repair for the mining industry, serum ferritin levels were evaluated in a completely different sample of 67 paired controls from a blood blank. The percentage of hyperferritinemia (>300 *μ*g/L) in this sample was 13.6%, which is similar to the value obtained from MD (Figure 1) (13.4%). These results are in agreement with a cross-sectional study of a population group from Mexico City [23], which found that the prevalence of high levels of ferritin (>300 *μ*g/L) was 12% in men and 4.8% in women [23].

On the other hand, when transferrin saturation was evaluated in the 66 volunteers by means of TIBC, only 4.5% of the population presented high levels of iron, and not one of the volunteers diagnosed with hyperferritinemia presented abnormal values of TIBC. These results indicate that high levels of ferritin found in our study are not related to hemochromatosis. This fact agrees with the extremely low prevalence of primary hemochromatosis in the Chilean population [24]. It has been estimated that the C282Y mutation of the HFE gene, in Chilean population, affects only 1 out of every 6250 people [24].

In order to determine the relationship between hyperferritinemia and metabolic syndrome, the prevalence of metabolic syndrome was evaluated in MD subjects only.

We found that 21.2% of the total population (*N* = 66) agreed with metabolic syndrome diagnostic criteria, according to the ATPIII definition [25]. These results are close to the national media of metabolic syndrome prevalence for Chilean males, which has been estimated to be 23.0 ± 1.3% [26]. Significant correlations were found between serum ferritin and several diagnosis parameters of metabolic syndrome, such as BMI, fasting glucose, triglycerides, and HDL cholesterol (Figure 2). When the mean value of serum ferritin was determined in terms of the numbers of metabolic syndrome risk factors, a continuous and significant increase was observed, indicating that the prevalence of hyperferritinemia was higher in subjects with metabolic syndrome.

It was found that serum ferritin correlates significantly and positively with at least two parameters related with oxidative stress, hepatic damage, and insulin resistance, in our sample (*N* = 66). This information points out the relevance of determining the levels of serum ferritin in the diagnostic of metabolic syndrome.

The relationship between dietary sources and hyperferritinemia was performed analyzing micronutrients in the food consumption pattern of our subjects. The dietary iron intake of the volunteers was more than two times the recommended amount. Furthermore, besides iron, there are other micronutrients with high intake, such as selenium, copper, phosphorous, sodium, riboflavin, thiamin, niacin, folic acid, and vitamin B12.

Interestingly, white bread (Table 3) showed the most important contribution in almost all the micronutrients with higher values, including iron. In Chile, wheat flour has been enriched with iron, riboflavin, niacin, and thiamin by legislation since 1950 [27], when modifications were introduced, fortifying wheat flour with folic acid with the aim of preventing anemia and neural tube defects, among other diseases [27]. Nevertheless, the success of this program has been questioned due to found evidence that associates folic acid fortification with an additional risk of colon cancer [28].

Despite the high contribution of white bread to the total iron intake of MD volunteers, no significant correlations were found with serum ferritin concentration. These results indicate that iron supplementation of white bread is not responsible for the hyperferritinemia found in MD volunteers. These results are in agreement with the low absorption of inorganic iron, reported in subjects with serum ferritin concentration ≥ 60 *μ*g/L [29]. This fact was also found in male subjects with BMI greater than 27 kg/m^2^ and levels of serum ferritin greater than 400 *μ*g/L, where intestinal absorption of the stable isotope Fe^58^ was even lower than overweight controls with normal levels of serum ferritin [30].

Significant correlation was established between ferritin concentration and red meat consumption, which was the second source of iron most important in our study. Recently, in a cross-sectional study, an association between red meat intake and inflammation, as well as an elevated risk of metabolic syndrome, was reported in women [12]. Further studies are required to confirm this finding and to determine whether the bioavailability of heme iron increases in subjects under some conditions and how this could contribute to the development of metabolic syndrome.

Given the information presented in this work, we confirm the results reported previously showing that high serum ferritin concentration is associated with metabolic syndrome and with oxidative stress markers and indicating the importance of evaluating serum ferritin in the diagnostic of metabolic syndrome. We present evidence that suggests that high intake of all kinds of red meat could be responsible for hyperferritinemia.

## Figures and Tables

**Table 1:** Anthropometric, clinical, and biochemical parameters evaluated for the 66 volunteers.

Parameters	Total volunteers (*n* 66)		Reference values
	Mean	SD	
Anthropometric parameters			
Age (years)	44.8	11.5	
Weight (kg)	82.7	7.8	
BMI (kg/m^2^)	27.9	2.9	18.5–26.5
Waist circumference (cm)	96.6	10.1	<102
Clinical parameters			
Systolic BP (mmHg)	119.6	8.3	90–119
Diastolic BP (mmHg)	70.3	8.9	60–79
Biochemical parameters			
Total cholesterol (mg/dL)	199.2	39.6	<200
HDL (mg/dL)	46.1	10.9	>40
LDL (mg/dL)	123.1	28.4	<130
Triglycerides (mg/dL)	151.3	86.3	<150
Serum ferritin (*μ*g/L)	321.8	235.3	12–300
Total iron binding capacity (*μ*g/dL)	303.5	33.5	240–450
Erythrocytes	5.1	0.3	4.2–5.9
Hematocrits (%)	45.8	2.3	40.7–50.3
Glucose (mg/dL)	91.3	20.8	82–110
Hemoglobin (g/dL)	15.5	0.8	13.8–18.0
HOMA	3.5	2.3	≤3.8
Blood insulin (*μ*U/mL)	15.0	6.5	5–25

**Table 2:** Correlation between serum ferritin concentration and oxidative stress markers, hepatic damage, insulin resistance, and inflammation response.

Parameter	Pearson coefficient (*r*)	*P* value
Oxidative stress		
Carbonyl group (nmol/mg protein)	0.320	0.009
AOPP (*μ*mol/L)	0.388	0.001
Glycated HB (%)	0.247	0.048
Hepatic damage		
GGT (U/L)	0.155	0.001
SGOT (U/L)	0.170	<0.001
Insulin resistance		
HOMA	0.447	<0.001
Blood insulin	0.264	0.034
Fasting glucose	0.481	<0.001
Inflammation		
C-reactive protein	−0.067	0.6096
Fibrinogen	−0.081	0.5408
Leukocytes	−0.166	0.1827

**Table 3:** Daily dietary intake of micronutrients for Maestranza Diesel workers. The data correspond to the mean value results given by the food processor of surveys registered by three consecutive days.

Source	Minerals			Vitamins				
	Fe (%)	Na (%)	Se (%)	Riboflavin (%)	Thiamin (%)	B12 (%)	Niacin (%)	Folic acid (%)
Red meat^*^	14.5	13.9	25.5	16.4	5.4	50.6	32.6	4.4
White meat	2.9	4.5	5.2	5.5	1.5	4.1	11.5	4.6
Cereals	6.39	6.89	8.4	4.8	10.2	0	4.1	21.6
Legumes	2.7	0.9	0.3	0.8	1.2	0	0.29	7.1
Fish and seafood	4.4	5.2	35.1	2.3	1.0	35.1	13.9	1.7
White bread	56.6	52.1	41.8	60.7	73.3	10.2	33.9	47.9
Vegetables	12.4	16.5	1.4	9.5	7.4	0	3.6	16.2

^*^All kinds of red meat including processed red meat.
